# Vitamin B12 responsive developmental and epileptic encephalopathy due to a novel mutation in the *FUT2* gene: a case report

**DOI:** 10.1186/s12887-024-05106-1

**Published:** 2024-09-30

**Authors:** PKBUC Bandara, Wasana Wijenayake, Sanjaya Fernando, Padmapani Padeniya, Sachith Mettananda

**Affiliations:** 1 Colombo North Teaching Hospital, Ragama, Sri Lanka; 2 Department of Anatomy, Faculty of Medicine, University of Kelaniya, Ragama, Sri Lanka; 3 Department of Paediatrics, Faculty of Medicine, University of Kelaniya, Thalagolla Road, Ragama, 11010 Sri Lanka

**Keywords:** Epilepsy, Epileptic encephalopathy, FUT2, Infantile spasms, Vitamin B12, Vitamin-responsive epileptic encephalopathies

## Abstract

**Background:**

Vitamin B12 deficiency is a recognised cause of neurological manifestations, including peripheral neuropathy, behavioural changes, and seizures. However, developmental and epileptic encephalopathy due to vitamin B12 deficiency is very rare. Here, we report an infant with vitamin B12-responsive developmental and epileptic encephalopathy due to a novel mutation in the fucosyltransferase 2 (*FUT2)* gene responsible for vitamin B12 absorption.

**Case presentation:**

An 11-month-old girl of non-consanguineous parents presented with recurrent episodes of seizures since four months. Her seizures started as flexor epileptic spasms occurring in clusters resembling infantile epileptic spasms syndrome with hypsarrhythmia in the electroencephalogram. She was treated with multiple drugs, including high-dose prednisolone, vigabatrin, sodium valproate, levetiracetam and clobazam, without any response, and she continued to have seizures at 11 months. She had an early developmental delay with maximally achieving partial head control and responsive smile at four months. Her development regressed with the onset of seizure; at 11 months, her developmental age was below six weeks. On examination, she was pale and had generalised hypotonia with normal muscle power and reflexes.

Her full blood count and blood picture revealed macrocytic anaemia with oval and round macrocytes. Bone marrow aspiration showed hypercellular marrow erythropoiesis with normoblastic and megaloblastic maturation. Due to the unusual association of refractory epilepsy and megaloblastic anaemia, a rare genetic disease of the vitamin B12 or folate pathways was suspected. The whole exome sequencing revealed a homozygous missense variant in exon 2 of the *FUT2* gene associated with reduced vitamin B12 absorption and low plasma vitamin B12 levels, confirming the diagnosis of vitamin B12 deficiency related developmental and epileptic encephalopathy. She was started on intramuscular hydroxocobalamin, for which she showed a marked response with reduced seizure frequency.

**Conclusion:**

We report a novel variant in the *FUT2* gene associated with vitamin B12-responsive developmental and epileptic encephalopathy and megaloblastic anaemia. This case report highlights the importance of timely genetic testing in children with refractory developmental and epileptic encephalopathy to identify treatable causes.

## Background

Developmental and epileptic encephalopathy (DEE) is a rare group of disorders presenting in infancy with severe early-onset epilepsy, in which the neurodevelopmental comorbidity may be attributable to both the underlying cause and the adverse effects of uncontrolled epileptic activity [1]. Vitamin B12 deficiency is a recognised cause of numerous neurological abnormalities, including sub-acute combined degeneration of the cord, peripheral neuropathy, mood and behavioural changes, cognitive decline, and seizures [2]. However, vitamin B12 deficiency causing DEE is very rare [3]. Here, we report a novel mutation in the fucosyltransferase 2 (*FUT2)* gene responsible for vitamin B12 absorption, causing vitamin B12-responsive DEE in an infant.

## Case presentation

**Fig. 1:**
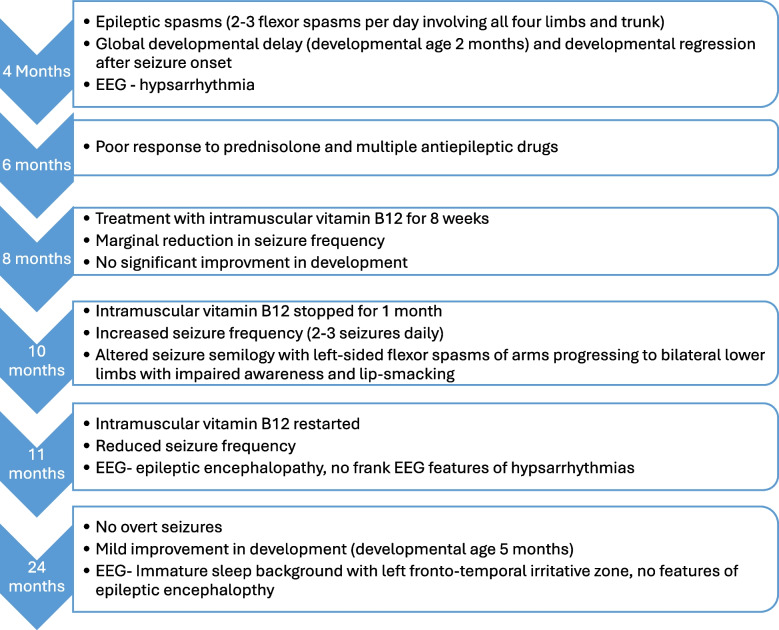
A flow diagram showing a summary of the clinical course and medical interventions

An 11-month-old girl presented with recurrent episodes of seizures since the age of four months. She is the third-born child to non-consanguineous parents without a family history of epilepsy. At four months of age, she developed repeated episodes of seizures manifested as flexor epileptic spasms of all four limbs and trunk. These epileptic spasms appeared in clusters at a frequency of two to three per day, each lasting five minutes. A clinical diagnosis of infantile epileptic spasms syndrome (IESS) had been made, and her electroencephalogram (EEG) at four months showed hypsarrhythmia supporting the diagnosis. She was commenced on oral prednisolone 10 mg 6 hourly for one week, which was later increased to 20 mg 8 hourly. She did not show a response to steroid therapy and had poor seizure control despite being on multiple antiepileptics that included vigabatrin, sodium valproate, levetiracetam and clobazam. She was also treated with a brief course of intramuscular vitamin B12 (1000 µg once daily for seven days followed by 1000 µg once a week for eight weeks) and multivitamin supplements containing folic acid, thiamine, pyridoxine and vitamin C. She showed a marginal improvement with reduced seizure frequency; however, vitamin B12 was stopped for one month due to the patient defaulting treatment. At 11 months, she continued to have seizures at a frequency of 2–3 per day (Fig. 1).

The girl had an early developmental delay with maximally achieving partial head control and responsive smile at four months of age. She then had developmental regression with the onset of seizures. At 11 months, she did not have head control, could not fix or follow and could not make cooing sounds. Her developmental age was less than six weeks. Her mother took folic acid supplementation and a balanced, nutritious, non-vegetarian diet during the antenatal period. The baby was exclusively breastfed for six months, after which weaning was started. Complementary food included animal proteins. She did not have features of malabsorption or inflammatory bowel disease.

On examination, the baby was pale but was not icteric. Her length and weight were within normal limits, and her head circumference was 43.5 cm (between -1SD and median). She did not have oedema, lymphadenopathy or hepatosplenomegaly. There were no neurocutaneous manifestations. Neurological examination revealed generalised hypotonia with normal muscle power and reflexes. Cranial nerves and cerebellar system were normal. Eye examination revealed features of cortico-visual impairment. Her cardiovascular and respiratory systems were clinically normal.

Her full blood count revealed haemoglobin- 7.9 g/dL (normal 11–15), mean corpuscular volume- 92fL (normal 70–90), mean corpuscular haemoglobin- 31.8 pg (normal 27–32), mean corpuscular haemoglobin concentration- 34.3 g/dL (normal 32–36), white cell count- 10.2 × 10^9^/L (normal 4–15 × 10^9^), and platelet count- 421 × 10^9^/L (normal 150–450 × 10^9^). Blood film showed reduced red cell number and normocytic normochromic cell population with oval and round macrocytes. Her serum ferritin was 110 ng/ml (normal 7–140), and the haemoglobin high-performance liquid chromatography did not reveal abnormal haemoglobin variants. Bone marrow aspiration revealed moderately hypercellular marrow erythropoiesis with normoblastic and megaloblastic maturation and relatively suppressed granulopoiesis. Biochemical investigations revealed serum vitamin B12- 759 pg/ml (normal 197–771), thyroid stimulating hormone- 3.84mIU/L (normal 0.45–4.5), red blood cell folate- 787.5 ng/ml (normal 121–651), homocysteine-3.08 µmol/L (normal < 10), methionine- 25 µmol/L (normal 14–48) and lactate-2.97 mmol/l (normal 2–4). Plasma amino acid and urine organic acid profiles were also normal. Magnetic Resonance Imaging of the brain showed mild bilateral frontal and anterior parietal lobe atrophy.

Due to the unusual association of DEE, refractory epilepsy and megaloblastic anaemia, a rare genetic disease of the vitamin B12 or folate pathways was suspected. A detailed genetic evaluation with whole exome sequencing was carried out in the index patient, and it revealed a novel homozygous missense variant in exon 2 of the *FUT2* gene (ENST00000425340.2/ NC_000019.10:g.48703291C > T). As per the American College of Medical Genetics and Genomics mutation classification guidelines, this mutation was classified as a variant of uncertain significance[4]. Yet, in silico analysis by Mutation tester and SIFT tools predicts that the mutation is deleterious and has a significant protein change. *FUT2* mutation is associated with reduced vitamin B12 absorption and low plasma vitamin B12 levels, thus confirming the diagnosis of vitamin B12 deficiency associated with DEE in the index patient. Genetic testing of parents was not done due to limitations of resources. The child was started on intramuscular hydroxocobalamin 1000 µg every other day for seven days, weekly for two months and monthly thereafter. At the 12-month post-treatment review, at the age of 2 years, she showed marked improvement with a considerable reduction in seizure frequency, and her serum vitamin B12 level was 2000 pg/ml (normal 197–771). We planned to continue vitamin B12 1000 µg one to three monthly.

## Discussion

Vitamin-responsive epileptic encephalopathy is an important treatable cause of DEE and drug-resistant epilepsy. It encompasses a broad group of disorders that include pyridoxine-dependent epilepsy, pyridoxal 5-phosphate-dependent epilepsy, folinic acid-responsive epilepsies, biotinidase deficiency and vitamin B12 deficiency [3].

Here, we report an infant with vitamin B12-responsive DEE due to a homozygous mutation in the *FUT2* gene. The infant initially had features of IESS with developmental delay and hypsarrhythmia in EEG. However, she did not respond to the standard treatment of IESS, which prompted us to consider genetic testing and to arrive at an accurate diagnosis. Although epileptic spasms are a very rare manifestation of vitamin B12 deficiency, refractory infantile spasms associated with hypsarrhythmia due to vitamin B12 deficiency have previously been reported in babies born to vitamin B12 deficient mothers [5–7]. Our report highlights the importance of investigating for vitamin B12 deficiency by doing a full blood count and vitamin B12 levels and performing genetic testing to rule out rare genetic causes of DEE when epileptic spasms are refractory to standard treatment of IESS.

Although we found a mutation in the vitamin B12 absorption pathway, the serum vitamin B12 level of the child done at presentation to our unit at 11 months was normal. This is likely because the child was initially treated with a short course of parenteral vitamin B12. Metabolic abnormalities of vitamin B12 deficiency are known to remain in some genetic causes of vitamin B12 deficiency even after the serum vitamin B12 levels are normalised [8]. The genetic confirmation of the homozygous mutation of the *FUT2* gene and the clinical response to optimal doses of intramuscular vitamin B12 confirmed the diagnosis of vitamin B12-responsive DEE in our child. There is one similar previous report of an infant developing IESS due to vitamin B12 deficiency while on treatment, even after normalization of the vitamin B12 levels [7].

The *FUT2* gene located in chromosome 19 encodes galactoside α-(1,2)-fucosyltransferase enzyme that regulates the expression of the blood group H-antigen on the surface of epithelial cells and body fluids [9]. Several variants of the *FUT2* gene are associated with reduced plasma vitamin B12 levels due to reduced absorption[10]. Although the exact mechanism of how *FUT2* gene mutation affects vitamin B12 absorption is unknown, variants of *FUT2* altering the susceptibility to *Helicobacter pylori* are reported [11]. The H-antigen encoded by *FUT2* has been described to mediate *Helicobacter pylori* attachment to human gastric mucosal cells, and *FUT2* variants are associated with reduced synthesis of intrinsic factor, which is vital for vitamin B12 absorption [10].

In conclusion, we report a novel variant in the *FUT2* gene associated with vitamin B12-responsive DDE and megaloblastic anaemia. This case report highlights the importance of timely genetic testing in children with refractory DDE to identify treatable causes.

## Data Availability

The data that support the findings of this study are not openly available due to reasons of sensitivity and are available from the corresponding author upon reasonable request.

## Abbreviations

DDE: Developmental and epileptic encephalopathy
EEG: Electroencephalogram
IESS: Infantile epileptic spasms syndrome
